# Interventions to reduce leprosy related stigma: A systematic review

**DOI:** 10.1371/journal.pgph.0003440

**Published:** 2024-08-22

**Authors:** Matthew Willis, Anil Fastenau, Srilekha Penna, Gonnie Klabbers

**Affiliations:** 1 Department of Health, Ethics and Society, Faculty of Health, Medicine and Life Sciences, Maastricht University, Maastricht, The Netherlands; 2 Marie Adelaide Leprosy Centre, Karachi, Pakistan; 3 German Leprosy and Tuberculosis Relief Association (GLRA/DAHW), Wurzburg, Germany; 4 Heidelberg Institute of Global Health, University of Heidelberg, Heidelberg, Germany; 5 Department of Global Health, Institute of Public Health and Nursing Research, University of Bremen, Bremen, Germany; Università degli Studi di Firenze: Universita degli Studi di Firenze, ITALY

## Abstract

Stigmatisation is a major issue faced by those affected by leprosy globally. Reducing stigmatisation encourages care seeking behaviour to occur earlier and can help reduce harm and spread of leprosy. This systematic literature review aimed to summarise what effective stigma reducing interventions exist for leprosy, and as a secondary question explore what evidence exists regarding their cost. A systematic literature review was conducted. Three databases–PubMed, Embase and Web of science–were searched using the search terms “leprosy”, “interven*”, “reduc*”, and “stigma*”.Seventeen publications were eligible for inclusion in the review. The current manuscript identified interventions under 6 main categories (i) Information, education, and communication (IEC) (ii) community led projects, (iii) Socioeconomic rehabilitation, (iv) mixed interventions, (v) integration of leprosy within the health system and (vi) Cosmetic or surgical care. Specific evidence regarding cost was only provided by one out of the seventeen papers. Multiple interventions were shown to successfully reduce leprosy related stigma, however, information on their cost is not readily available. The evidence uncovered by this review is restricted to three Asian countries; Nepal, India and Indonesia. To ensure the success of stigma reduction in leprosy interventions worldwide these interventions need to be tried in other leprosy endemic areas to test their effectiveness across contextual and cultural scenarios.

## Introduction

Leprosy stigma is a form of social stigma against those perceived to be affected by leprosy. Stigma is as old as the disease itself, with writings that date back to 2000 BC reporting on perceptions about those affected to be unclean, untrustworthy, and morally corrupt [1, 2]. More formal segregation and the formation of “leper colonies” became mainstream in 12th century Europe with the disease reaching pandemic levels after its introduction by those returning from the Crusades [3].

Infection with leprosy affects the nerves, skin, eyes, and lining of the nose. When the bacteria infect the nerves, they can become swollen under the skin. This can cause the affected areas to lose the ability to sense touch and pain, which can lead to injuries, like cuts and burns due to the loss of sensation. If left untreated this can lead to severe disfigurement of limbs due to the loss of pain reception and subsequent unintentional injury [4].

Leprosy stigma can be based on the perceived morality of those infected, cultural, and religious beliefs, fear of transmission and association with people considered “inferior” [5]. Stigma related to health conditions was defined by Erving Goffman as an attribute that signifies a difference or a deviation from “normal” and that in the extreme form a person is “less desirable, dangerous or weak” [6]. Today, in many countries where leprosy remains endemic (most new cases are in Asia and Africa, concentrated in low-income areas), affected individuals are still forced to live as outcasts, leading to a multitude of mental health issues associated with isolation and discrimination, thereby perpetuating a cycle of ill health [7]. In addition to personal isolation, community members still stigmatise those affected, with studies revealing that many community members would refuse to shake hands or share items with those affected [8]. Leprosy places a heavy economic burden on those affected as the associated stigma affects employment opportunities and subsequently culminates in a deterioration in economic status [9].

Classified as a neglected tropical disease (NTD), leprosy interventions are often underfunded and remain low priority on the global health agenda [10], despite leprosy being entirely treatable if detected in an early stage. However, the stigma still associated with the disease may prevent those affected from seeking treatment which in turn may exacerbate issues surrounding the allocation of sufficient funding and infrastructure to provide the necessary treatment [11]. Due to the low profile and underfunding associated with leprosy and other NTDs efforts to decrease the stigma associated with the disease are also neglected [12]. Addressing stigma may facilitate early help seeking, and by doing so promote early diagnosis and treatment. This systematic review seeks to examine what effective leprosy-stigma reducing interventions exist and gather available information about their cost.

The Health Stigma and discrimination framework articulates the stigmatisation process as it unfolds across what it refers to as the “socio-ecological” spectrum that exists in the community under study, the process is therefore context-specific [13]. The socio-ecological configurations give way to drivers and facilitators that determine stigmatisation manifestations, affecting health and well-being. Drivers and facilitators, Stigma marking, and stigma manifestations are subject to interventions, whereas the outcomes are subject to monitoring.

The framework captures the intersectionality of leprosy stigma in the sense that it recognises that the stigma associated with leprosy can often manifest itself in “marked stigma”, the perception of affected individuals being “unclean” [14]. In some countries the stigma facilitator of being viewed as “unclean” can intersect with other stigmatising factors in the culture such as in India where those of a lower caste have far higher rates of leprosy. This can lead to a “double blow” of stigmatisation whereby people are stigmatised due to their class and their disease status. Additionally, the cultural facilitator of gender perception, in countries with low levels of gender equality such as in many sub-Saharan countries can facilitate stigma [15, 16]. The result of this stigma is decreasing social participation, lower employment prospects, marital issues, and societal exclusion [17]. Due to the severe societal consequences of leprosy infection, there are often attempts by affected people to conceal their diagnosis–which can be considered as an outcome of stigma under the framework. This outcome leads to a delay in treatment, increased risk of severe disability and increased risk of community spread–the health and social impact of stigma in the framework [18]. Interventions to reduce stigma associated with leprosy aim to reduce the burden of the disease on those affected by removing psychological damage from isolation and to also facilitate openness and transparency so that people seek treatment without fear. A previous literature review had shown that interventions with some evidence of effectiveness in terms of stigma reduction comprise the integration of leprosy programmes into general health care; Information Education and Communication (IEC) programmes; and socio-economic rehabilitation programmes [19].

The current manuscript provides an updated perspective on the last decade following the previous review, thus incorporating new approaches aimed at mitigating stigma. It encompasses publications from all dates to comprehensively summarize the available evidence. Furthermore, the previous review overlooked inquiries into the cost implications of such interventions which we hope will provide useful evidence to future policymakers and researchers.

## Methods

### Design

A systematic review, following the PRISMA guidelines, was carried out to synthesise evidence on what stigma-reducing interventions exist for people affected by leprosy and to collate available information about the cost of these interventions.

### Eligibility criteria

All studies included were in English, there were no restrictions on publication dates or geographical location. Studies were only included where an intervention to address leprosy related stigma had been researched. This included qualitative, quantitative, and mixed method studies. The PICOS criteria [20] used for this study are: *Population*–Patients with diagnosed or suspected leprosy who may experience associated stigma; *Intervention*–an intervention designed to reduce stigma associated with leprosy/suspected leprosy; *Outcome*–reported stigma; *Comparison*–what did the researchers conclude regarding effectiveness and reported cost of strategies; and *Study type*–Qualitative, quantitative and mixed-methods studies.

### Search strategy

The automated search detailed below was completed in PubMed, Embase and Web of science with the string for each detailed in *Table 1*. The MeSh terms for PubMed were generated from 8 sample papers manually chosen as “relevant” to the study by the reviewers. Upon completion and conversion to other databases each search strand still returned all 8 “relevant papers”.

**Table 1:** Summary of search strategies.

Database: search 16/05/2023	Search item	Results returned
PubMed	("social behavior"[MeSH Terms] OR "stigma*"[Title/Abstract]) AND ("leprosy"[MeSH Terms] OR "leprosy"[Title/Abstract]) AND ("reduc*"[Title/Abstract] OR "intervention"[Title/Abstract])	153
Embase	(Exp social psychology/ OR stigma*. ti,ab,kf) AND (exp leprosy/ OR leprosy.ti,ab,kf) AND (reduc* OR interv*).ti,ab,kf	198
Web of Science	TS = (leprosy AND (stigma* or "social Behavior") AND (Reduc* OR intervention))	401

### Data collection and management

Data were maintained and managed using endnote referencing software. Results of the initial search were then reviewed using Rayyan [21]. Duplicates were both automatically and manually excluded. Two authors, MW and GK independently completed both title and abstract and full text screening, and discussed similarities and differences, after which consensus was reached by all authors as to which papers met inclusion criteria. The resultant articles were then reviewed independently by MW and GK, and information collated and tabulated on a standard form for all papers.

### Data extraction and analysis

Data extraction included setting, sample, study design, measures (stigma and outcome), effects and cost of intervention if available and is summarised in *S1 Appendix*. This was initially completed independently by MW and GK before a consensus was reached by all authors following discussion on what data to include. The health stigma and discrimination framework was used to evaluate each intervention in relation to the domains used by the framework.

### Risk of bias

In keeping with PRISMA guidelines each paper was screened for independent bias. CASP checklists were used to screen for any potential bias, this was carried out independently by MW and GK, with discrepancies then discussed and resolved. Results are summarised in *S2 Appendix* [22].

### Ethical considerations

Ethical clearance was obtained from Maastricht university and data collection commenced upon approval on the 16/05/2023.

## Results

**Fig 1:**
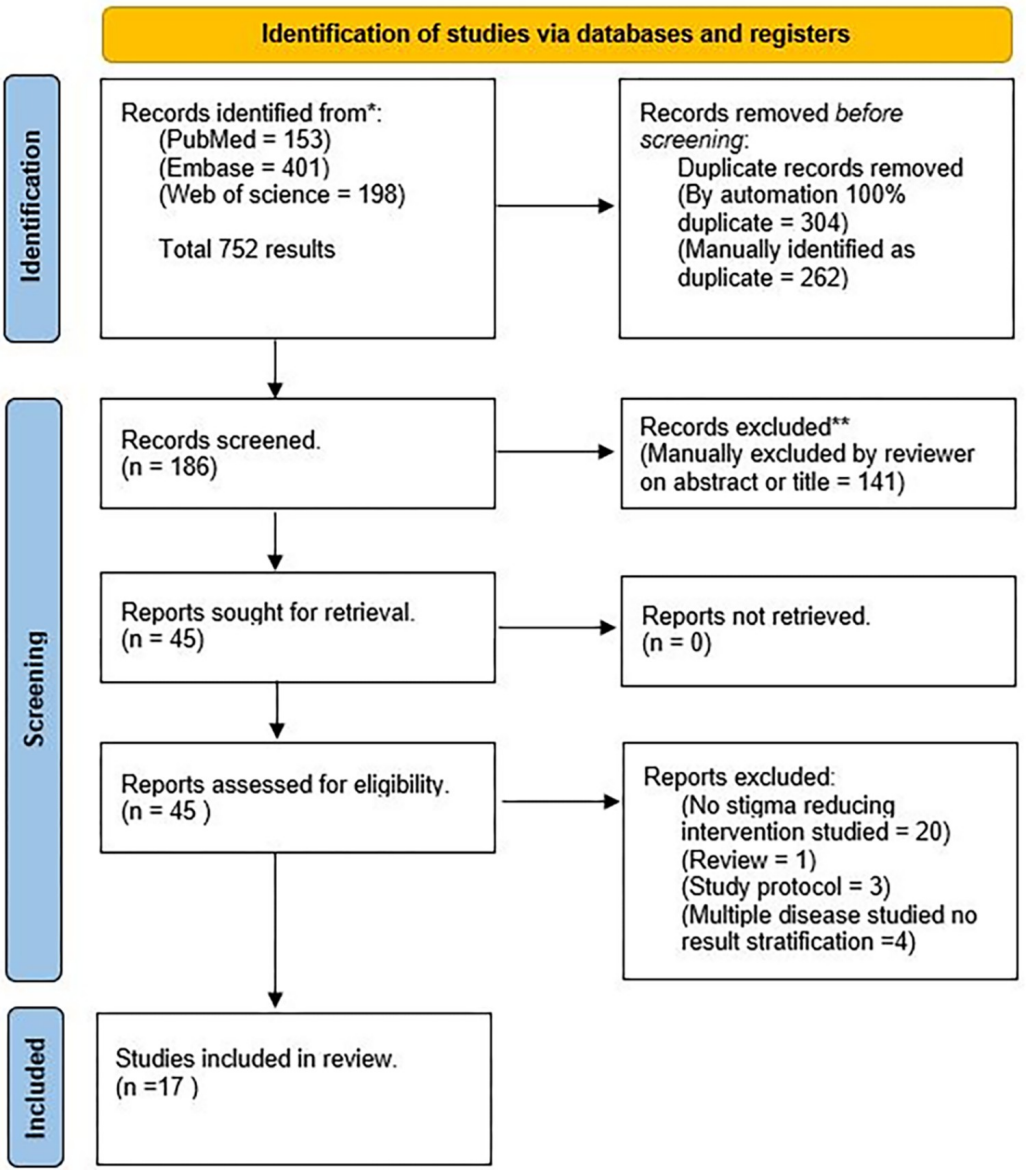
Summary of selection process for papers.

The initial database search identified 752 papers. After de-duplication (removing 506 papers), title and abstract screening (removing 141 papers), and full-text screening of 45 papers, 17 papers were found eligible for inclusion in this analysis (*Fig 1)*

The 17 papers included contain data from only 3 Asian countries, India (n = 8), Indonesia (n = 7) and Nepal (n = 2). The papers discussed interventions in different states, for India: Telangana (n = 2), Odisha (n = 1), Uttar Pradesh (n = 1), Maharashtra (n = 1), Tamil Nadu (n = 1) and multi state studies (n = 2); for Indonesia two provinces were represented: West Java (n = 6) and North Sulawesi (n = 1); and the two papers studying populations in Nepal were from the southern region of the country. The papers reviewed are listed with reference, paper title, study methodology/design, country/region, intervention, population/sample, setting, social, cultural, and political context, outcome, and information available about cost, in *S1 Appendix* [23–39].

The stigma reducing interventions identified by the review can be divided into 6 categories (i) Information, education, and communication (IEC) (ii) community led projects, (iii) Socioeconomic rehabilitation, (iv) mixed interventions, (v) integration of leprosy within the health system and (vi) Cosmetic or surgical care.

### (I) Information, education, and communication

Information, education, and communication interventions were studied in 7 papers across three countries: India (n = 3), (Nepal n = 2) and Indonesia (n = 2) [23, 27–30, 32, 38]. This category encompasses counselling, social skills training, awareness and empowerment training, resilience training and participatory video production.

Counselling was studied by two papers in Indonesia in West Java. The intervention focused on using lay or peer counsellors to counsel leprosy patients under the SARI (Stigma Assessment and Reduction of Impact) programme. The effect of lay and peer counsellors on perceived stigma was examined in qualitative focus groups. Patients were found to express that counselling from peers (those also currently or previously affected by leprosy) reduced stigma more than with lay counsellors by allowing those affected to empathise with their counsellor [29]. A cluster randomised control examining counselling also found significant decreases in SSS (self-stigma score) (SSS is a 9-item questionnaire with each question scoring 1–4 providing a maximum score of 36 and a minimum of 9) from 21.55 pre intervention to 12.00 after counselling (p <0.001) [32]. This intervention included patients with a WHO leprosy disability grade of 0–2 and integrated three types of counselling (individual, family, and group) into a 2-year programme which provided awareness of rights (healthcare, education). The counselling was delivered by both lay and peer counsellors and the decrease in SSS was reported post the two-year intervention.

Social skills training was studied by one paper in Tamil Nadu, India. The intervention provided 5 patients affected by leprosy with 10 day-long group-sessions of social skills training which involved identification of emotions and concerns of patients when interacting socially and analysis of positive and negative social interactions. The study included 3 patients from upper castes and two from lower castes, none of those involved were below the state poverty line. One participant was a Muslim and two each were Christian and Hindu respectively. Role-plays and videos, as well as live models were used. Post intervention patients expressed greater “acceptance, hope, courage and increased self-awareness” [23].

Specific information education and communication packages were studied by two papers in Nepal and one in India [28, 30, 38]. The outcomes of the P-Scale (participation scale) in the Stigma elimination programme (STEP) in Nepal did present substantial support to suggest that leprosy related stigma had been overcome in communities where STEP had been implemented [38]. STEP was a programme that sought to change the self-perception of patients as “victims” to a perception of positive empowerment–this change was driven by “facilitators” in each village and the results were based on STEP programmes in ten villages in southern Nepal, one of the poorest regions of the country. In Uttar Pradesh, India, another IEC intervention found the proportion of the population holding stigmatising views about those with leprosy fell from 86% to 61% post intervention with contextualised IEC [28]. This IEC package was delivered door to door by community workers and was provided pre-chemoprophylaxis. In Southern Nepal another programme that provided group-based education on leprosy and leprosy care found that 35% of the change in stigma related harm post intervention could be explained by the introduction of the IEC package [30].

In a dual state pilot study in India individual and family resilience training was found to explain 16% of the increase in resilience in Odisha and 24% in Telangana. The 10-week family-based intervention was designed to strengthen the resilience of individuals and families by improving their protective abilities and capacity to overcome adversity. The study found this effect on resilience scores was greater for both Hindus and males [27].

Participatory video production as a form of communication and education was studied—the intervention focused on the effect specifically on the producers of the videos (the stigmatised). Producing videos was found to lead to a “greater sense of togetherness” and a “greater willingness for action in the community” by the video-makers when explored via qualitative interviews in West Java, Indonesia [36].

### (II) Community led projects

Three papers studied community led projects to reduce stigma with all using a qualitative methodology and as such only qualitative data was found to support their use. In Bihar, India, Sustainable community-based rehabilitation for leprosy patients was trialled to reduce stigma. Patients stated, “their lives are much better after they came to Little Flower Hospital and that they now have dignity in life” [26]. In North Sulawesi, Indonesia, the LFV (leprosy friendly village) model was studied through interviews with those living there. The LFV model specifically aims to change the opinions of influential community members (religious leaders, village leaders, community health workers, schoolteachers, traditional healers, and other organizational leaders such as representatives from women groups, youth organizations etc.) to be more accommodating to leprosy affected people. Participants in the LFV reported perceiving less stigma and receiving better acceptance from their community members post implementation [25]. A tri-state study of, Uttar Pradesh, West Bengal, Chhattisgarh, using participatory action research investigated a community-based approach toward stigma reduction compared to control villages. This approach involved the creation of stigma reduction committees chaired by an influential local person. Researchers reported that the interventions were well accepted by the community and that patients reported a reduction in personal restrictions [39].

### (III) Socioeconomic rehabilitation

The evidence base for socioeconomic interventions alone is based on one paper from West Java in Indonesia which involved mainstreaming people affected by leprosy into existing microfinance businesses while simultaneously creating alternative microfinancing options–this was coined the “twin-track” approach. The randomised control trial found that the SSS showed a decrease of 8.5 points between pre- and post-intervention (p-value 0.004), the PSS (Participation Scale—Short) (13 questions scored from 0–4 giving a range of 0–52) a decrease of 3.6 points (p-value 0.0074) and the WHOQOL-BREF (WHO Quality of Life BREF) (26 item instrument with responses transformed to a scale of 0–100) an improvement of 4.3 (p-value 0.130) [33].

### (IV) Mixed interventions

Two papers researched interventions that can be considered a mix of at least 2 of the other themes outlined [34, 35].

One paper in West Java, Indonesia, studied the effect of contact intervention in two stages firstly the integration of people into the community and secondly educational material to address any misconceptions. The interview data showed that knowledge about leprosy increased, and that negative attitude reduced. The participants reported increased quality of life as evidenced by the adjusted mean total score of the EMIC-CSS (Explanatory Model Interview Catalogue Community Stigma Scale) (15 questions scored from 0–2 giving range of 0–30) reducing by 4.95 points among respondents who had attended a contact event (n = 58; p <0.001, effect size = 0.75) compared to the score at baseline (n = 213); and by the SDS (Social distance scale) (7 item questionnaire scored from 0–3 giving range of 0–21) scoring at 3.56 (p <0.001, effect size = 0.81). About 75% of those attending a contact event said they shared the information with others (median 10 persons) [34].

Socioeconomic interventions combined with peer counselling education were studied in a RCT in West Java, Indonesia. Among those affected significant differences in the reduction of stigma and participation restrictions were recorded. Social distance and social stigma were also significantly reduced among non-affected community members as well. Two of the five instruments (SSS, SDS, PSS, EMIC-CSS, WHOQOL- BREF) also changed in the control area, but these changes were far larger in the intervention areas [35]. The researchers proposed this could be due to training and economic interventions having an effect outside intervention areas or due to wider societal change.

### (V) Integration of leprosy care within the health system

Evidence regarding the merit of integration efforts were scarce with only one paper studying the impact of integrating leprosy services compared to a vertical approach in Maharashtra state India. Leprosy is treated as a vertical disease in India except for the states of Tamil Nadu and some parts of Maharashtra. The difference between a vertical and integrated approach is that in a vertical approach only designated staff and organisations deal with leprosy whereas in the integrated intervention studied this responsibility for leprosy treatment is held by the whole health system. The study explored the difference between villages involved in a community rural health project which integrates leprosy care and those who did not. The results indicated that stigma was minimal in the integrated areas when compared to the vertical areas. However, both control and integrated villages had a disproportionately high divorce rate amongst people married to a partner affected by leprosy which the authors posited could imply that hidden stigma is still prevalent in integrated areas [31].

### (VI) Cosmetic or surgical care

Evidence regarding the use of cosmetic or surgical interventions was based on two papers. The use of cosmetic camouflage, which involved applying makeup to visible lesions, was studied in Telangana, India. Both papers found reductions in stigma, however, neither sample was randomised nor were the cohorts large. Cosmetic camouflage was found to reduce DQLI (dermatology quality of life index) (DLQI consists of 10 questions concerning patients’ perception of the impact of skin diseases each scored between 0–4 giving a range of 0–40) from 16.67 ± 3.87 and to 2.23 ± 1.16 after its use, the difference (14.67 ± 3.87) was found to be statistically highly significant (p < 0.0001) [24]. Surgical correction of deformities to make them less visible were studied in Odisha, India. The research found that the proportion of people “very satisfied” with societal acceptance increased from 1% to 51% of patients post reconstructive surgery when surveyed [37].

### Cost of these approaches

Information regarding cost was extremely limited within the papers studied. Five papers made claims about their intervention being “low cost” or “inexpensive” without providing figures to backup these claims [28, 30, 32, 34, 35]. One paper posited that the use of microcredit as a form of financing should contribute to the sustainability of the project [33]. While only one paper provided definitive costing of their intervention with the cosmetic intervention costing $10 USD per month per patient at the time of publication [24].

### Methodological quality

The methodological quality of the papers included are summarised in *S2 Appendix.* Papers were evaluated using the CASP checklists–for mixed method studies papers are evaluated under both the Qualitative and RCT evaluation checklists. One paper was identified as a Cohort study and was analysed as such using the respective checklist.

Overall, the results found that the methodological quality of the evidence was generally quite high for each class of intervention. However, in some categories the methodological quality standard of evidence was more modest. This was particularly the case for integration of leprosy within the healthcare setting with only one paper present to be evaluated and only four out of ten CASP questions obtaining a definitive “yes”.

## Discussion

This review set out to examine what stigma-reducing interventions for leprosy affected individuals exist, whilst also synthesising available data about their cost. The current manuscript identified interventions under 6 main categories (i) Information, education, and communication (IEC) (ii) Community led projects, (iii) Socioeconomic rehabilitation, (iv) Mixed interventions, (v) Integration of leprosy within the health system and (vi) Cosmetic or surgical care. Cost was only reported in one of seventeen papers.

Although leprosy is unevenly distributed globally with Brazil, Indonesia, and India accounting for almost 80% of new cases [40], the evidence found and analysed by the current review was entirely limited to the continent of Asia. Furthermore, within the above mentioned three countries cases are often focalised within specific communities, with pockets of high endemicity present within certain sub- district levels. The absence of a breadth of countries and regions with the research limits the cultural and contextual evidence base for the interventions. Of the 6 classes of intervention analysed by this review only IEC and community-based interventions contained papers from more than one country (IEC had papers from India, Indonesia and Nepal, and Community based interventions had an intervention studied in India and Indonesia).

Most interventions targeted those affected by the stigma and did not target communities and societal beliefs that stigmatise. This could be interpreted as victim blaming and could be criticised as a bias within the research field toward models of stigmatisation that ignore the complexity of stigma within different cultures [41]. Only two of the studies showed multi aspect interventions, specifically including the communities in which affected individuals live. If future interventions target community attitudes as well as individual perception and resilience it could lead to more permanent change according to the framework by removing “drivers” and “facilitators”. Another observation was that six of the papers under review, 35% of the evidence presented [23–26, 29] had publication dates within the last 3 years. This is a promising sign that impetus is growing to further study stigma reduction interventions.—as the review included all publication years. Evidence regarding cost was sparse with five papers making claims about their intervention being “low cost” or “inexpensive” without providing figures to backup these claims [28, 30, 32, 34, 35] and only one paper providing costing per patient [24]. Using cost, and subsequently calculated cost-effectiveness, as a metric for interventions is seen as an ethical dilemma within medical research as on one hand it is a means by which to ensure the best use of budgets, but scholars also argue it can be unethical and lead to patients receiving inferior treatment in the name of “cost-effectiveness” [42, 43].

However, costing of interventions does provide a means by which policy makers can see if an intervention is feasible for their budget and by comparing cost with effectiveness limited budgets can be used to the best extent while extra funding is sought by affected communities [44]. Horton, Gelband et al. conducted a systematic review of 93 health interventions targeting major infectious diseases (including Leprosy, TB, malaria, and NTDs) in low- and middle-income countries (LMICs) spanning from 2005 to 2006, subsequently ranking them based on their cost-effectiveness. They argued for the utility of cost-effectiveness rankings in informing national healthcare planning and budget allocation. Acknowledging the dynamic nature of NTD priorities, they note that advancements in technology, changes in behaviour, and fluctuations in the prices of vaccinations and treatment drugs have altered the priorities since 2006. They additionally advocate for regularly updating cost data. This analysis underscores the scarcity of evidence regarding the expenses associated with interventions targeting leprosy stigma. Consequently, this poses challenges when allocating budgets towards programs aimed at reducing stigma [45].

Not all authors provide specifics on the context in which the interventions took place. Most studies took place in low-income areas and within communities of Asian ethnicity, religiously most participants identified as either Muslim or Hindu, however data were lacking on Christian and folk religions, and on non-religious. The lack of geographical and cultural diversity in the papers highlights the need for further studies to evaluate the effectiveness of these tools in other areas. Although the review does include data from three highly leprosy endemic hotspots it does not account for countries in South America or Africa where there is also a large burden of disease [40]. In addition to missing these “high endemic” countries the papers also do not include any data pertaining to “low endemic” regions of countries where leprosy is often even lower on the health agenda and has the potential to be just as marginalising or even more so [46].

### Strengths and limitations

This review covers all evidence published on leprosy stigma reducing interventions, and it is the first study to systematically collate information available about the cost of these interventions. Possibly, relevant papers were not included because of not being included in the databases searched, because of being published in different languages than English, or by not being available in full text. This could be a reason for the lack of geographical and cultural diversity presented in the studied interventions.

## Conclusion

The results of this systematic review highlight the fact that successful interventions to reduce leprosy associated stigma exist. The review highlights the need for more research into interventions in different cultural settings and highlights the fact that evidence on the cost of interventions is severely limited and recommends further research to compare interventions in this regard. The review also raises the observation that most existing interventions target the individual affected by stigma and not the stigmatisers in the community, future research should not only seek to reduce the impact of stigma on those affected but also intervene to reduce stigmatising community views and beliefs. Overall, further research and impetus into the field of stigma reducing interventions in leprosy will aid future elimination programmes and help achieve the WHO’s “Toward zero leprosy 2030” goal [47].

### Recommendations

Stigma reduction interventions should be included in future strategies to combat leprosy within communities. The stigma reduction interventions summarised above are shown to improve wellbeing and perception of wellbeing amongst those affected by leprosy across all studies included in this review. The interventions encourage patients to seek care earlier not only increasing positive treatment outcomes for themselves but also reducing the spread of leprosy in the community.

Future interventions should build on and consider the positive findings of previous interventions. Future comprehensive interventions should combine the reduction of stigma within communities while also reducing internalised stigma and increasing empowerment, socioeconomic status, and self-perception in affected individuals.

Future studies should incorporate contextualisation of stigma reduction interventions The review only presents evidence for three countries in Asia. To successfully provide a means by which to tackle leprosy related stigma globally interventions must be tested and evaluated in more countries and communities to expand the evidence base and provide solutions by which to culturally adapt interventions. Related to this, the findings of the present study need to be validated in low-endemic regions as well.

Finally, future studies should include information on cost which can then be extrapolated to cost-effectiveness measures between interventions, allowing policymakers to evaluate whether an intervention is feasible for their region. Although the authors do not want to suggest that cost be a starting point for policymakers, we feel that evidence around cost would allow for the calculation of cost-effectiveness, facilitating stakeholders to best utilise current budgets while continuing to campaign for more funding into stigma-reduction interventions.

## Supporting information

S1 Appendix Results table—summary of findings in each paper. (DOCX)

S2 Appendix Methodological checklist -summary of CASP checklists for each paper included in study. (DOCX)

S3 Appendix PRISMA checklist. (DOCX)
