# Supplementary material for: Interventions to reduce leprosy related stigma: A systematic review
Source: PLOS Glob Public Health. 2024 Aug 22;4(8):e0003440. doi: 10.1371/journal.pgph.0003440 (PMC11340997; doi:10.1371/journal.pgph.0003440)
Supplement: S1 Appendix — (DOCX) [file pgph.0003440.s001.docx]

| Author and title | Study methodology/design | Country/region | Intervention | Population/sample | Setting | Social, cultural, and political context | Outcome | Information about cost |
| --- | --- | --- | --- | --- | --- | --- | --- | --- |
| Effectiveness of social skills training for reduction of self-perceived stigma in leprosy patients in rural India –  A preliminary study  Augustine V.; Longmore M.; Richard M.E | Semi-structured qualitative interviews. | Tamil Nadu, India | The participants attended 10 days of group-sessions that taught social skills training over 3 weeks. The social skills training programme was adapted from a social rehabilitation-training programme devised for head and neck cancer patients. | Five male leprosy inpatients of similar socioeconomic background (aged between 21 and 56). Four of the five had between 5 or 6 years of formal education. And one had several years’ experience in business. | Inpatient hospital | None of the participants were below the local poverty line. Three were upper caste and two lower castes. The participants consisted of one Muslim, two Hindus and two Christians.  Four had a farming background and one was a hairdresser. | Outcomes were assessed through pre and post intervention interviews. Following the intervention patients expressed a greater sense of “acceptance, hope, courage and increased self-awareness”.  These results suggested that the social-skills training caused an increase in self- esteem, as seen through changes in self-perception and behaviour. | No information provided |
| Cosmetic camouflage of visible skin lesions enhances life quality indices in leprosy as in vitiligo patients: An effective stigma reduction strategy.   Narasimha Raoa P.; Vellalab M.; Potharajuc A.R.; Udaya Kirand K | Quantitative based on calculation of DLQI (dermatologic life quality index) | Department of Dermatology, Bhaskar Medical College, Yenkapally, RR district, Telangana, India | Cosmetic camouflaged was applied to skin lesions using Dermacolor®. Patients followed up for 6 months with DLQI assessed at beginning and end of study period | 9 patients with leprosy (male 5; female 4), with skin patches on the face and forearm, were included in the study. The ages of the patients ranged from 16 to 47 years, with a mean age of 28.9 years. Borderline Tuberculoid (BT) leprosy in 6 (66.6%) patients, followed by Tuberculoid (TT) in 2 and Indeterminate leprosy in 1 patient. | Outpatients in Telangana, India | All the leprosy patients in the study accepted cosmetic camouflage readily. | Overall, the total mean DLQI of leprosy patients before the use of camouflage was 16.67 ± 3.87 and reduced to 2.23 ± 1.16 after its use, the difference (14.67 ± 3.87) was statistically highly significant (p < 0.0001). | $10 USD a month per patient |
| "We are not afraid anymore..." Capturing the most significant change of the Leprosy Friendly Village approach in North Sulawesi, Indonesia  Budiawan T.; Ferdiana A.; Daendel S.; Widayati R.; de Hart J.; Soesman M.; Mieras L.; | Qualitative interviews - Data collected using in-depth interviews (IDIs) and focus group discussions (FGDs) according to the most significant change (MSC) technique | North Minahasa district in North Sulawesi, Indonesia | The Leprosy Friendly Village (LFV) approach was initiated in 2012 and designed to improve leprosy case finding in North Minahasa District in North Sulawesi Province especially in highly endemic villages. The LFV approach was specifically aimed at improving the attitude of village influential community members (ICM) and all HC staff towards leprosy. | Data were collected from all 15 villages where the LFV (leprosy friendly village) approach was implemented.  28 interviews with patients, community members and health care workers - 20 female 8 male. | Leprosy friendly village in a rural part of Indonesia with a high endemicity of leprosy.  case detection rate of 11 per 100,000 population. It consists of 120 villages with a total population of 157,918 in 2012. | Study stated, it was not easy to find persons affected by leprosy who were willing to speak out. Thus, there were in total 7 persons affected by leprosy recruited as ICM in the 15 villages where the LFV approach was implemented. | The most significant change in the community was less discrimination and fear of leprosy, more referral of suspects to HCs and better community integration of persons affected by leprosy, who perceived less stigma and better acceptance from the community. | No information available |
| Patients help other patients: Qualitative study on a longstanding community cooperative to tackle leprosy in India.  Jung S.H.; Han H.W.; Koh H.; Yu S.-Y.; Nawa N.; Morita A.; Ong K.I.C.; Jimba M.; Oh J. AO - Yu, Soo-Young | Grounded Theory using semi-structured interviews | Raxaul, Bihar, India | Sustainable community-based rehabilitation for leprosy patients. Interviews about experiences living in the little flower hospital community. | Participants recruited in three groups. Patients, workers, and board members.  Group 1 contained 11.  Group 2 contained 13.  Group 3 contained 4 | Little flower hospital community. Which is a leprosy hospital with a “leper colony” of 1000 people living around it. They use community-based rehabilitation to help people live with leprosy. | Bihar is one of the poorest districts in India, where one fifth of the whole leprosy patients of India have been forming an endemic colony | The findings presented suggested that the sustainable development model of LFHC. Improve certain patient outcomes with patients suggesting, that “their lives are much better after they came to LFH and that they now have dignity in life” | No information available |
| Strengthening individual and family resilience against leprosy-related discrimination: A pilot intervention study  Van'T Noordende A.T.; Pereira Z.B.D.S.; Biswas P.; Ilyas M.; Krishnan V.; Parasa J.; Kuipers P.; | Quasi-experimental, mixed methods data collection. | Two sites – urban areas in Telangana state and rural areas in Odisha state | Intervention tried to strengthen resilience by using ten weekly sessions in a family-based format. Each session adopted an action learning and problem-solving approach to the activities design. In addition, a separate session about rights was taught. | 80 participants were included in the study. Over half of the participants (n = 41, 51%) were from urban areas in Telangana state. The median age of the participants was 35 years. Half of the participants had not had any (formal) education (n = 40, 50%). About one third had paid work (n = 24, 30%), and a third were unemployed (n = 23, 29%). While all participants in Odisha state were Hindu (n = 39, 100%), the participants in Telangana state were either Muslim (n = 21, 51%) or Hindu (n = 20, 49%). | In 2019, the prevalence of leprosy was 1.45 per 10,000 population in Odisha state and 0.62 per 10,000 population in Telangana state (State NLEP report, 2019 | Diversity of participants included in terms of language, rural vs urban and native language. | Odisha – model explained 16% of the increase in resilience. Men had significantly more improvement. Those with employment listed other significantly less.  Telangana  Model explained 24% of improvement in resilience scores. Those who were Hindu experienced significantly greater improvements. (50% increase explainable.) | No information available |
| Changing perception and improving knowledge of leprosy: An intervention study in Uttar Pradesh, India  van 't Noordende AT; Lisam S; Singh V; Sadiq A; Agarwal A; Hinders DC; Richardus JH; van Brakel WH; Korfage IJ; | Mixed methods – pre/post intervention study design | Two districts in Uttar Pradesh, northern India: Chandauli (population 1.95 million, 1548 villages) and Fatehpur (population 2.63 million, 1476 villages). | Interventions provide contextualized (adapted to the local context) information, education and communication (IEC) and are implemented before the implementation of the actual chemoprophylaxis | 1067 participants were included in the first survey and 843 participants in the second survey | These districts have a relatively high number of new leprosy patients annually with a new case detection rate of 5.9 per 100,000 population in both Chandauli and Fatehpur, in March 2019 (District Leprosy Office | Programme administered by door-door ASHA workers. | In Survey 1, 86% of participants had stigmatizing attitudes towards leprosy on the EMIC-CSS (n = 747; 86% of the contacts and community members, 84% of the health workers). In Survey 2, this was 61% (n = 393; 65% of the contacts and community members, 43% of the health workers). | No specific details paper states the interventions are “relatively low cost” |
| Lay and peer counsellors to reduce leprosy-related stigma--lessons learnt in Cirebon, Indonesia.  Lusli M; Peters RM; Zweekhorst MB; Van Brakel WH; Seda FS; Bunders JF; Irwanto | Qualitative Group discussions | Kabupaten, Cirebon (Cirebon District) which is in the province of West-Java near the provincial border with Central-Java. Indonesia | Exploration of involving lay and peer counsellors in the reduction of stigma and its impact in the field of leprosy. It is based on the perspectives of the lay and peer counsellors of the SARI project. The data was collected between April 2012 and March 2013. | 198 people affected by leprosy were offered counselling by the 11 lay and 12 peer counsellors; 145 accepted this offer. The other 53 either did not need counselling or did not want to participate for example due to worries about disclosure. | The SARI project had 23 counsellors. They were made up of11 lay counsellors (six of whom were physically or visually disabled and five non-disabled, with the 12 peer counsellors being those affected by leprosy. | Participant councillors learned about how to facilitate counselling during 14 days of training (4 hours of training per day, so 56 hours in total) | Study concluded using lay and peer counsellors was effective. Though peer counsellors were reported as better reducing stigma by allowing people to empathise with their counsellor. | No information |
| Health literacy among self-help leprosy group members reduces stereotype endorsement and stigma-related harm in rural Nepal.  Muldoon OT; Jay S; O'Donnell AT; Winterburn M; Moynihan AB; O'Connell BH; Choudhary R; Jha K; Sah AK; | Quantitative data collection scoring stigma using SARI scale to grade stigma before and after intervention. | In ten villages in four districts of Southern Nepal: Dhanusha, Mahottari, Sarlahi and Sindhuli | Group-based community support to improve their leprosy self-care and general health literacy. | 71 people affected by leprosy | The villages are in Terai, which ranks lowest in Nepal's National Human Development Indicators. It scores more poorly than other regions of Nepal on indicators such as employment, life expectancy, literacy, maternal mortality. | This region has some of highest rates of leprosy prevalence and disability in South Asia, at approximately 2.76 per 10,000 population in 2019 compared with 2015 global prevalence of 0.18 per 10,000 population. | There was a significant direct effect of self-help group belonging change on T2 stigma-related harm, β = −0.48, p = 0.006.  T2 stigma-related harm improvement via health literacy change and T2 stereotyped endorsement was significant (Indirect effects, β = −0.06; 95% CI [−0.18, −0.008]) explaining 35% of the variance in T2 stigma-related harm | No specific details but the intervention is described – “community-based approach to management of leprosy is not only cost-efficient” |
| Social stigma: a comparative qualitative study of integrated and vertical care approaches to leprosy.  Arole S; Premkumar R; Arole R; Maury M; Saunderson P; | Qualitative comparison between vertical and integrated health approaches. In depth analysis of interviews and a frequency chart of coherent answers. | Three villages in Maharashtra state India | Vertical vs integrated approach for leprosy care | Data was collected in three areas. The first two areas using the integrated approach and the third using a vertical approach.  Overall, there were 14 integrated patients interviewed vs 10 vertical. | The prevalence rate of leprosy in Maharashtra (1999-2000) was 3.7 per 100,000.  2.64 in the integrated PHC approach area and 4.7 in the vertical area | The integrated and vertical were similar in terms of caste religion language SEC and geographical terrain. | The results indicate stigma was minimal in the integrated areas when compared to the vertical areas. However, both control and integrated villages had a disproportionately high divorce rate – implying masked stigma may remain | No information on cost of each intervention. |
| The Impact of a Rights-Based Counselling Intervention to Reduce Stigma in People Affected by Leprosy in Indonesia.  Lusli M; Peters R; van Brakel W; Zweekhorst M; Iancu S; Bunders J; Irwanto; Regeer B; | Mixed methods (three scales, interviews, focus group discussions and reflection notes)  SARI project is a cluster-randomized controlled intervention study | Cirebon District, Indonesia | Rights based counselling delivered by lay or peer counsellors. | 67 counselling clients | Patients with disability ranging from grade 0-2. Majority had primary or secondary education. | Participant councillors learned about how to facilitate counselling during 14 days of training (4 hours of training per day, so 56 hours in total) | Qualitative data indicates that knowledge and rights trigger change in stigmatization.  Total scores of SSS, PSS and WHOQOL-BREF. The analysis of the SSS, PSS and WHOQOL-BREF data shows that there are large and significant differences between the total scores at baseline and at the final survey in the counselling clients. The SSS total reduced from 21.55 to 12.00 (p-value <0.001) | No specific information but the study states the following “During the design of the counselling intervention costs were considered, as we realised that a costly intervention would not be sustainable in a context were resources and time are scarce. In general, the execution of the intervention is inexpensive because of the involvement of lay and peer counsellors who live near the clients. Some costs are involved, and some time investments need to be made for example for the training and supervision of the lay and peer counsellors” |
| Assessing the Impact of the Twin Track Socio-Economic Intervention on Reducing Leprosy-Related Stigma in Cirebon District, Indonesia.  Dadun D; Peters RMH; van Brakel WH; Bunders JGF; Irwanto I; Regeer BJ; | Randomized-controlled trial mixed-methods study design was used to test the effectiveness of the SED intervention. | Cirebon District, Indonesia | Twin track intervention of mainstreaming people affected into existing microfinance businesses while also creating alternative microfinancing options.  Three scales were used to measure stigma and participation restrictions among 30 SED clients and 57 controls, 20 in-depth interviews with SED clients and seven Focus Group Discussions (FGDs) with key persons were held and 65 profiles of the clients were written up and analysed. | 369 people were involved in the different SED-related activities, including 110 affected by leprosy, 251 community members and eight persons with a disability. In total, 66 persons affected by leprosy accessed 71 units of microcredit | Rural villages in Cirebon Indonesia | People affected by leprosy had expressed that they felt insecure about obtaining credit from a formal institution. | The analysis shows a significant reduction in the SSS and PSS total scores of SED participants between the baseline assessment and the final survey. The SSS showed a decrease of 8.5 points between pre- and post-intervention (p-value 0.004), the PSS a decrease of 3.6 points (p-value 0.0074) and the WHOQOL-BREF an improvement of 4.3 (p-value 0.130) | No information – however, the paper states that the use of microcredit in community should in theory contribute to the sustainability of a project |
| A Cluster-Randomized Controlled Intervention Study to Assess the Effect of a Contact Intervention in Reducing Leprosy-Related Stigma in Indonesia.  Peters RM; Dadun; Zweekhorst MB; Bunders JF; Irwanto; van Brakel WH; | Cluster randomised control intervention  Mixed methods used to evaluate outcome | Cirebon District, Indonesia | Contact intervention – two key phases.  Phase 1 contact between affected persons and the public – for example the creation of storytelling material to express people with leprosy’s stories to the public.  Secondly education which seeks to address misconceptions. | 91 contact events were organised in 62 villages, directly reaching 4,443 community members (mean 49 per event).  Of which 213 in baseline survey and 375 in final survey. | Contact interventions were spread throughout Cirebon district – map available | In initial study they found a diversity of responses ranging from “I don’t know” to more neutral or factual responses as leprosy is a “skin disease” and “contagious disease”. In addition, some responses were value laden, for example, leprosy is “worrisome”, “horrible”, “disgusting”, “extremely grievous” and “a curse” | The interview data showed that knowledge about leprosy increased, and that negative attitude reduced. The adjusted mean total score  of the EMIC-CSS reduced by 4.95 points among respondents who had attended a contact  event (n = 58; p <0.001, effect size = 0.75) compared to the score at baseline (n = 213); for  the SDS this was 3.56 (p <0.001, effect size = 0.81). About 75% of those attending a contact  event said they shared the information with others (median 10 persons) | No direct information states – “does not require expensive technology” |
| Impact of socio-economic development, contact and peer counselling on stigma against persons affected by leprosy in Cirebon, Indonesia –a randomised controlled trial.  Dadun D; Van Brakel WH; Peters RMH; Lusli M; Zweekhorst MBM; Bunders JGF; Irwanto; | Randomised control trial with Mixed methods to analyse results | Cirebon district, Indonesia | Testing combined impact of socio-economic development, contact counselling and peer counselling. | Two study populations: people affected by leprosy (under treatment or released from treatment up to 3 years earlier) and community members. We decided to follow people affected by leprosy over time (cohort) and to have two different cross-sectional observations of community members.  237 in intervention cohort. | Study states Cirebon district was included due to “its high level of leprosy-related stigma as perceived by experts, and no initiatives to address this” | Only included patients who spoke the Bahasa language | Among affected people (n = 237), significant differences in reduction of stigma and participation restrictions were found in all intervention areas and an improvement in quality of life in some intervention areas. Social distance and social stigma significantly reduced among community members (n =213 and 375) in the two intervention areas where the contact intervention was implemented. Two of the five instruments indicated changes in the control area, but the changes in the intervention areas were much larger. | No specific details but describes intervention as “low cost”. |
| People like me don't make things like that': Participatory video as a method for reducing leprosy-related stigma.  Peters RM; Zweekhorst MB; van Brakel WH; Bunders JF; Irwanto; | Qualitative interviews with video makers and participants observing. | Cirebon district, Indonesia | Use of participatory video creation by those affected by stigma to reduce stigma felt by the video producers | 8 for the first video  4 for the second (only those with visual impairments) | The first 8 had no visible deformities due to stigma while the second group of 4 did. In both cases 11 were approached to create videos. | Reasons for declining were work obligations outside town, harvest season, unforeseen problems (fire) at the plantation, unexpected family situation that needed attention, old age and poor health, pregnancy, one mother’s hesitations because her son was shy and a husband’s denial of permission for his wife to participate. | The impact on participants ranged from having a good time to a greater sense of togetherness, increased self-esteem, individual agency and willingness to take action in the community. | No information provided |
| Role of Reconstructive Surgery (RCS) in Improving the Quality of Life of Leprosy Afflicted Persons.  Lenka D; Mahapatra A; | A semi-structured questionnaire was administered to assess their understanding of better quality of life (QOL) after reconstructive surgery. | Sonepur district of Odisha, India | Reconstructive surgery to improve appearance and functionality of affected body parts | 60 patients who had undergone reconstructive surgery | As this was based surgical outcome only patients with visible deformities were included. | The subjects were highly dispersed geographically within the district. Limitation as some interviews had to be cut short | Percentage who deemed themselves “very satisfied” with societal acceptance increased from 1% to 51% post RCS | No information available |
| STEP: an intervention to address the issue of stigma related to leprosy in Southern Nepal.  Cross H; Choudhary R; | Quantitate application of “p-scale survey” and interviews. Non random sample used for comparison | Southern Nepal | Stigma elimination programme (STEP), STEP was a programme that sought to change the self-perception of patients as “victims” to a perception of positive empowerment – this change was driven by “facilitators” in each village | 152 people affected by leprosy | All subjects were from rural, agricultural environments. Due to the limitations of curfew the control villages were nearby non-step villages reachable before curfew. | A curfew was in place at the time of study due to Maoist insurgents limiting study participation | The outcomes of the P Scale investigation did present substantial support to suggest that leprosy related stigma had been overcome in communities where STEP had been implemented. | No specific information available |
| A study on community-based approaches to reduce leprosy stigma in India.  Raju MS; Rao PS; Mutatkar RK; | Qualitative Participatory Action Research, community based | India, 3 states, Uttar Pradesh, west Bengal, Chhattisgarh | Impact of taking a community-based approach toward stigma reduction compared to control village 60 stigma reduction organising committees (SROCs) in 3 states were formed, each led by a chair (community leader figure) consisting of community members (with and without leprosy), who first were informed about leprosy, onset, consequences, and treatment by healthcare professionals, then set out to develop activities to implement in the community. Best practices were studied. | 60 SROCs were formed, consisting of varying numbers of members (16 small committees 8-10 members; 39 medium sized groups 11-15 members; and 5 big committees 16-20 members) serving their communities in 2 rural blocks in each of the 3 states | in 2 rural blocks in each of the 3 states | Not further specified/identified | SROC interventions well accepted by the communities. Variety of interventions, providing counselling and moral support to leprosy afflicted people and family members, education, referrals to clinic, use of videos, commonly included. Some addressed leprosy inflicted only, other activities the whole community.  All activities were supervised and observed, and informal feedback obtained, Changes in pat restrictions reported on by family members. Formal evaluation is yet to take place | No information provided |
