# Supplementary material for: Interventions to reduce leprosy related stigma: A systematic review
Source: PLOS Glob Public Health. 2024 Aug 22;4(8):e0003440. doi: 10.1371/journal.pgph.0003440 (PMC11340997; doi:10.1371/journal.pgph.0003440)
Supplement: S2 Appendix — (DOCX) [file pgph.0003440.s002.docx]

# Appendix 2 – Methodological Assessment

Key:

Green=Yes

Red=No

Yellow=Can’t tell

All relevant questions for each table can be found below.

**CASP – Qualitative**

| Reference | Q.1 | Q.2 | Q.3 | Q.4 | Q.5 | Q.6 | Q.7 | Q.8 | Q.9 | Q.10 |
| --- | --- | --- | --- | --- | --- | --- | --- | --- | --- | --- |
| Augustine V, Longmore M, Ebenezer M, Richard J. Effectiveness of Social Skills Training for reduction of self-perceived Stigma in Leprosy Patients in rural India – a preliminary study. Leprosy Review. 2012 Mar 1;83(1):80–92. |  |  |  |  |  |  |  |  |  |  |
| Budiawan T, Ferdiana A, Daendel S, Widayati R, de Hart J, Soesman M, et al. “We are not afraid anymore…” Capturing the most significant change of the Leprosy Friendly Village approach in North Sulawesi, Indonesia. Leprosy Review. 2020 Jun 1;91(2):173–89. |  |  |  |  |  |  |  |  |  |  |
| Jung SH, Han HW, Koh H, Yu SY, Nawa N, Morita A, et al. Patients help other patients: Qualitative study on a longstanding community cooperative to tackle leprosy in India. PLoS neglected tropical diseases [Internet]. 2020 Jan 1 [cited 2023 Apr 19];14(1):e0008016. Available from: <https://pubmed.ncbi.nlm.nih.gov/31929530/> |  |  |  |  |  |  |  |  |  |  |
| van’t Noordende AT, Bakirtzief da Silva Pereira Z, Biswas P, Ilyas M, Krishnan V, Parasa J, et al. Strengthening individual and family resilience against leprosy-related discrimination: A pilot intervention study. Nardi SMT, editor. PLOS Neglected Tropical Diseases. 2021 Apr 2;15(4):e0009329. |  |  |  |  |  |  |  |  |  |  |
| van ‘t Noordende AT, Lisam S, Singh V, Sadiq A, Agarwal A, Hinders DC, et al. Changing perception and improving knowledge of leprosy: An intervention study in Uttar Pradesh, India. Chandler DJ, editor. PLOS Neglected Tropical Diseases [Internet]. 2021 Aug 23 [cited 2023 Feb 2];15(8):e0009654. Available from: https://www.ncbi.nlm.nih.gov/pmc/articles/PMC8412405/ |  |  |  |  |  |  |  |  |  |  |
| Lusli M, Peters RMH, Zweekhorst MBM, Van Brakel WH, Seda FSSE, Bunders JFG, et al. Lay and peer counsellors to reduce leprosy-related stigma – lessons learnt in Cirebon, Indonesia. Leprosy Review. 2015 Mar 1;86(1):37–53. |  |  |  |  |  |  |  |  |  |  |
| Arole S, Premkumar R, Arole R, Maury M, Saunderson P. Social stigma: a comparative qualitative study of integrated and vertical care approaches to leprosy. Leprosy Review. 2002 Jun 1;73(2):186–96. |  |  |  |  |  |  |  |  |  |  |
| Peters R  M. H., Zweekhorst M  B. M., van Brakel WH, Bunders J  F. G., Irwanto. “People like me don’t make things like that”: Participatory video as a method for reducing leprosy-related stigma. Global Public Health. 2016 Mar 28;11(5-6):666–82. |  |  |  |  |  |  |  |  |  |  |
| Lenka, D, and A Mahapatra. “Role of Reconstructive Surgery (RCS) in Improving the Quality of Life of Leprosy Afflicted Persons.” Indian journal of leprosy vol. 2016 88,1: 7-12. |  |  |  |  |  |  |  |  |  |  |
| Cross H, Choudhary R. STEP: An intervention to address the issue of stigma related to leprosy in Southern Nepal. Leprosy Review. 2005 Dec 1;76(4):316–24. |  |  |  |  |  |  |  |  |  |  |

**Questions - CASP Qualitative**

1. Was there a clear statement of the aims of the research?
2. Was there a clear statement of the aims of the research?
3. Was the research design appropriate to address the aims of the research?
4. Was the recruitment strategy appropriate to the aims of the research?
5. Was the data collected in a way that addressed the research issue?
6. Has the relationship between researcher and participants been adequately considered?
7. Have ethical issues been taken into consideration?
8. Was the data analysis sufficiently rigorous?
9. Is there a clear statement of findings?
10. How valuable is the research?

**CASP - RCT**

| Paper | Q.1 | Q.2 | Q.3 | Q.4 | Q.5 | Q.6 | Q.7 | Q.8 | Q.9 | Q.10 | Q.11 |
| --- | --- | --- | --- | --- | --- | --- | --- | --- | --- | --- | --- |
| Narasimha Rao P, Vellala M, Potharaju AR, Udaya Kiran K. Cosmetic camouflage of visible skin lesions enhances life quality indices in leprosy as in vitiligo patients: an effective stigma reduction strategy. Leprosy Review. 2020 Dec 1;91(4):343–52 |  |  |  |  |  |  |  |  |  |  |  |
| van’t Noordende AT, Bakirtzief da Silva Pereira Z, Biswas P, Ilyas M, Krishnan V, Parasa J, et al. Strengthening individual and family resilience against leprosy-related discrimination: A pilot intervention study. Nardi SMT, editor. PLOS Neglected Tropical Diseases. 2021 Apr 2;15(4):e0009329. |  |  |  |  |  |  |  |  |  |  |  |
| van ‘t Noordende AT, Lisam S, Singh V, Sadiq A, Agarwal A, Hinders DC, et al. Changing perception and improving knowledge of leprosy: An intervention study in Uttar Pradesh, India. Chandler DJ, editor. PLOS Neglected Tropical Diseases [Internet]. 2021 Aug 23 [cited 2023 Feb 2];15(8):e0009654. Available from: https://www.ncbi.nlm.nih.gov/pmc/articles/PMC8412405/ |  |  |  |  |  |  |  |  |  |  |  |
| Lusli M, Peters R, van Brakel W, Zweekhorst M, Iancu S, Bunders J, et al. The Impact of a Rights-Based Counselling Intervention to Reduce Stigma in People Affected by Leprosy in Indonesia. Johnson C, editor. PLOS Neglected Tropical Diseases [Internet]. 2016 Dec 13 [cited 2019 May 19];10(12):e0005088. Available from: https://www.ncbi.nlm.nih.gov/pmc/articles/PMC5154499/ |  |  |  |  |  |  |  |  |  |  |  |
| Dadun D, Peters R, van Brakel W, Bunders J, Irwanto I, Regeer B. Assessing the Impact of the Twin Track Socio-Economic Intervention on Reducing Leprosy-Related Stigma in Cirebon District, Indonesia. International Journal of Environmental Research and Public Health. 2019 Jan 26;16(3):349. |  |  |  |  |  |  |  |  |  |  |  |
| Peters RMH, Dadun, Zweekhorst MBM, Bunders JFG, Irwanto, van Brakel WH. A Cluster-Randomized Controlled Intervention Study to Assess the Effect of a Contact Intervention in Reducing Leprosy-Related Stigma in Indonesia. Phillips RO, editor. PLOS Neglected Tropical Diseases. 2015 Oct 20;9(10):e0004003. |  |  |  |  |  |  |  |  |  |  |  |
| Dadun D, Van Brakel WH, Peters RMH, Lusli M, Zweekhorst MBM, Bunders JGF, et al. Impact of socio-economic development, contact and peer counselling on stigma against persons affected by leprosy in Cirebon, Indonesia – a randomised controlled trial. Leprosy Review. 2017 Mar 1;88(1):2–22. |  |  |  |  |  |  |  |  |  |  |  |
| Cross H, Choudhary R. STEP: An intervention to address the issue of stigma related to leprosy in Southern Nepal. Leprosy Review. 2005 Dec 1;76(4):316–24. |  |  |  |  |  |  |  |  |  |  |  |

**Questions - CASP RCT**

1. Did the study address a clearly focused research question?
2. Was the assignment of participants to interventions randomised?
3. Were all participants who entered the study accounted for at its conclusion?
4. Were the participants ‘blind’ to intervention they were given? Were the investigators ‘blind’ to the intervention they were giving to participants? Were the people assessing/analysing outcome/s ‘blinded’?
5. Were the study groups similar at the start of the randomised controlled trial?
6. Apart from the experimental intervention, did each study group receive the same level of care (that is, were they treated equally)?
7. Were the effects of intervention reported comprehensively?
8. Was the precision of the estimate of the intervention or treatment effect reported?
9. Do the benefits of the experimental intervention outweigh the harms and costs?
10. Can the results be applied to your local population/in your context?
11. Would the experimental intervention provide greater value to the people in your care than any of the existing interventions?

**CASP - Cohort Study**

| Reference | Q.1 | Q.2 | Q.3 | Q.4 | Q.5 | Q.6 | Q.7 | Q.8 | Q.9 | Q.10 | Q.11 | Q.12 |
| --- | --- | --- | --- | --- | --- | --- | --- | --- | --- | --- | --- | --- |
| Muldoon OT, Jay S, O’Donnell AT, Winterburn M, Moynihan AB, O’Connell BH, et al. Health literacy among self‐help leprosy group members reduces stereotype endorsement and stigma‐related harm in rural Nepal. Health & Social Care in the Community. 2022 Feb 27; |  |  |  |  |  |  |  |  |  |  |  |  |

**Questions – CASP Cohort Study**

1. Did the study address a clearly focused issue?
2. Was the cohort recruited in an acceptable way?
3. Was the exposure accurately measured to minimise bias?
4. Was the outcome accurately measured to minimise bias?
5. Have the authors identified all important confounding factors? Have they taken account of the confounding factors in the design and/or analysis?
6. Was the follow up of subjects complete enough? Was the follow up of subjects long enough?
7. What are the results of this study?
8. How precise are the results?
9. Do you believe the results?
10. Can the results be applied to the local population?
11. Do the results of this study fit with other available evidence?
12. What are the implications of this study for practice?
